# Short-form video reward expectancy, study-session fragmentation, and self-reported deep-work impairment among King Saud University students: a cross-sectional study

**DOI:** 10.3389/fpsyg.2026.1969859

**Published:** 2026-10-30

**Authors:** Musaad Bedah Alsahly, Abdulaziz Khaled Sahhari, Eyad Hussain M. Alzubaidi, Rayan Saleh Alguhaiz, Rayan Hassan Alshehri, Ali Anwar Aljammah, Abdullah Mazen Alansari, Thamer Abdullah Alsufayan, Thamir Al-khlaiwi

**Affiliations:** Department of Physiology, College of Medicine, King Saud University, Riyadh, Saudi Arabia

**Keywords:** academic focus, deep-work impairment, reward expectancy, Saudi Arabia, short-form videos, study-session fragmentation, university students

## Abstract

**Background and objective:**

Short-form video (SFV) platforms are widely used among university students and provide brief, personalized, and rewarding content. This may encourage repeated scrolling, frequent phone checking, and difficulty maintaining sustained academic focus. This study aimed to describe the proportion of King Saud University students reporting high SFV reward expectancy in this sample, and to examine its association with study-session fragmentation and self-reported deep-work impairment.

**Methods:**

A cross-sectional analytical study was conducted among students at King Saud University, Riyadh, Saudi Arabia. Data on SFV reward expectancy, study-session fragmentation, and deep-work impairment were collected using a structured electronic questionnaire. Pearson's and Spearman's correlations were used for bivariate analysis, and hierarchical multiple linear regression was used to estimate associations adjusted for demographic, academic, and device-use covariates, with standardized coefficients, 95% confidence intervals, collinearity diagnostics, and residual checks reported.

**Results:**

A total of 514 students were included in the analysis. High SFV reward expectancy was reported by 58.4% of respondents (95% CI: 54.1–62.6%). SFV reward expectancy was positively correlated with study-session fragmentation (*r* = 0.646, 95% CI 0.593–0.694, *p* < 0.001) and with deep-work impairment (*r* = 0.518, 95% CI 0.452–0.578, *p* < 0.001). In fully adjusted models, each one-point increase in SFV reward expectancy was associated with a 0.441-point higher fragmentation score (95% CI 0.379–0.503, β = 0.499, *p* < 0.001) and a 0.357-point higher deep-work impairment score (95% CI 0.288–0.426, β = 0.431, *p* < 0.001). Associations were attenuated but remained statistically significant after adjustment.

**Conclusion:**

High SFV reward expectancy was commonly reported in this sample and was significantly associated with greater study-session fragmentation and greater self-reported deep-work impairment, independent of measured demographic, academic, and device-use characteristics. Because the design is cross-sectional and all measures were self-reported, these findings are associative and cannot establish temporal order or causation. These findings highlight the need for longitudinal and intervention research using objective measures to evaluate the underlying mechanisms and to inform strategies promoting healthier technology use among university students.

## Introduction

Short-form videos (SFV) platforms available through social media, including YouTube Shorts, Instagram Reels, and TikTok have become a major part of daily digital life among university students (Jain et al., 2025). These platforms are designed around brief, highly engaging, and algorithmically personalized videos that encourage continuous scrolling and repeated viewing (Zhao and Wagner, 2022; Zhao, 2021). Unlike traditional forms of media consumption, SFV platforms provide rapid shifts in content, immediate entertainment, and frequent emotional or social rewards, making them especially appealing to young adults (Zhao and Wagner, 2022; Zhao, 2021; Ye et al., 2022). As university students increasingly rely on smartphones and digital platforms throughout the day, concerns have grown regarding the potential association between SFV use and attention, multitasking, academic focus, and study behavior (Alruwaili, 2025; Hsieh, 2025; Xie et al., 2023). Among the proposed mechanisms underlying these associations, SFV reward expectancy has emerged as an important concept in explaining repetitive SFV engagement and its potential relationship with study-session fragmentation and deep-work impairment.

SFV reward expectancy refers to the expectation that watching SFVs will provide quick pleasure, entertainment, relief, or social validation, encouraging repeated scrolling and prolonged engagement with this type of content (Xie et al., 2023; Yan et al., 2024; Sun et al., 2022). This reward-based pattern may make it more difficult for students to remain engaged in demanding academic tasks. Studying often requires sustained attention, delayed gratification, and resistance to distraction, whereas SFV platforms offer immediate stimulation and effortless content switching (Yan et al., 2024; Chen et al., 2022). Over time, frequent exposure to rapid digital rewards may be associated with fragmented study habits, frequent phone checking, and a reduced ability to maintain uninterrupted focus (Hsieh, 2025; Xie et al., 2023; Yan et al., 2024; Chen et al., 2022). Previous research has linked problematic SFV use with cognitive and academic concerns. Studies have reported associations between excessive SFV use and reduced attention, impaired inhibitory control, memory difficulties, cognitive fatigue, and lower academic engagement (Ye et al., 2022; Yan et al., 2024; Sun et al., 2022; Chen et al., 2022; Ye et al., 2023). Collectively, these cognitive and behavioral patterns may accompany study-session fragmentation and difficulty maintaining continuous academic engagement.

Study-session fragmentation refers to repeated study interruptions through behaviors such as phone checking, multitasking, and switching between academic tasks and digital content. In the context of SFV platforms, this fragmentation may be encouraged by design features such as personalized recommendations, algorithmic content delivery, and continuous scrolling, which increase immersion and make users more likely to return repeatedly (Jain et al., 2025; Zhao and Wagner, 2022). Neurocognitive evidence suggests that personalized SFV content can activate reward-related brain regions, including the ventral tegmental area and default mode network, and that short-video addiction has been associated with neuroanatomical and functional brain differences (Su et al., 2021; Gao et al., 2025). These neurocognitive findings derive from separate experimental and imaging studies; no neurocognitive or imaging measures were obtained in the present study, and they are cited here only as candidate mechanisms motivating the present research question.

Closely related to study-session fragmentation is the concept of deep work, popularized by Newport as the ability to sustain undistracted concentration on cognitively demanding tasks. Although the term originates outside the psychological literature, it maps closely onto established constructs of sustained attention, attentional control, and the regulation of cognitive effort (Newport, 2016; Liu et al., 2022); in the current study, it is operationalized as self-reported difficulty sustaining concentration on academic work, and is therefore referred to throughout as deep-work impairment rather than deep-work capacity. This difficulty may be related to procrastination, reduced attentional control, academic anxiety, and lower engagement (Xie et al., 2023). Evidence supports this concern, as smartphone use frequency has been negatively associated with academic performance (Hsieh, 2025; Li, 2024; Ma et al., 2025).

Existing research has predominantly focused on general SFV exposure, problematic use, or addiction-like behavior, whereas comparatively limited attention has been given to SFV reward expectancy as a distinct behavioral and cognitive construct (Alruwaili, 2025; Xie et al., 2023; Yan et al., 2024). Furthermore, few studies have explored how SFV reward expectancy relates to study-session fragmentation, including interruptions, multitasking, and repeated switching between academic tasks and digital content. Even less is known about its potential association with self-reported deep-work impairment and sustained academic focus among university students. Addressing these gaps is particularly important within the Saudi university context, where smartphone accessibility and social media engagement are highly prevalent (Alosaimi et al., 2016). Therefore, the present study aimed to examine the association between SFV reward expectancy and both study-session fragmentation and deep-work impairment among King Saud University students in Riyadh, Saudi Arabia, and to describe the proportion of students in this sample reporting high SFV reward expectancy.

## Methodology

### Study design, setting, and participants

This cross-sectional analytical study was conducted among students at King Saud University, Riyadh, Saudi Arabia. The overall project ran from September 2025 to April 2026. Following approval by the Institutional Review Board on 14 December 2025, participant recruitment and data collection took place between December 2025 and April 2026; no data were collected before ethical approval was granted. Inclusion criteria involved all male and female students currently enrolled at King Saud University across all colleges and academic years, who agreed to participate and provided informed consent. Participants who submitted incomplete questionnaires or did not provide informed consent or failed to meet the eligibility criteria were excluded from the final analysis.

Participants were recruited through official King Saud University communication channels, including the official university email system (Tawasul), supplemented by student WhatsApp groups and direct in-person recruitment across different colleges and halls. Interested students accessed the online questionnaire via Google Forms. Although the approved protocol specified stratified random sampling across colleges, this proved unfeasible because the university communication system does not permit college-stratified sampling frames or the tracking of individual invitations. Recruitment therefore proceeded as an open, university-wide voluntary-response (non-probability) survey. This deviation is clearly reported because it limits the interpretation of the descriptive proportions reported below, which summarize this sample only and are not population prevalence estimates for King Saud University or for Saudi university students generally.

Participant flow was as follows. A total of 515 questionnaire submissions were received, all of which recorded consent and confirmed eligibility as currently enrolled King Saud University students. During data cleaning for the present revision, one record was identified as an exact duplicate of the immediately preceding submission across all 49 recorded variables, including all 28 Likert-scale items, and was removed as a probable double submission. No submission contained missing data on any analyzed variable, because the electronic form required a response to every item before submission; therefore, no participant was excluded for incompleteness and no imputation was required. The final analytic sample included 514 participants.

Because invitations were distributed through an open university-wide mailing list, WhatsApp groups, and in-person approach, the total number of students who received or opened the invitation was not recorded, and a conventional response rate therefore cannot be computed. This limitation is reported in the Limitations section.

### Instrument and scale scoring

The questionnaire collected information on sociodemographic and academic characteristics, short-form video use patterns, and the three study scales. With the limited availability of validated instruments presenting SFV reward expectancy and deep work, items were modified from previously published instruments examining attention, cognitive control, mind-wandering, attentional control, and behavioral effects of short-video and digital-media use (Ye et al., 2022; Li, 2024; Liu, 2022; Mrazek et al., 2013; Derryberry and Reed, 2002; Newport, 2016; Liu et al., 2022). The instrument was pilot-tested for clarity and suitability before deployment; pilot responses were not included in the final analytic sample. The full instrument is provided as Supplementary File 1.

All three scales used a five-point Likert response format (1 = strongly disagree to 5 = strongly agree). The SFV reward expectancy scale included 8 items, the study-session fragmentation scale 11 items, and the deep-work impairment scale 9 items. Each scale score was computed as the mean of its constituent items, yielding a possible range of 1.00 to 5.00, with higher scores indicating greater reward expectancy, greater fragmentation, and greater deep-work impairment, respectively. Internal consistency was excellent for SFV reward expectancy (α = 0.906), high for study-session fragmentation (α = 0.891), and good for deep-work impairment (α = 0.855).

Scale scores were additionally categorized as low, medium, or high using equal thirds of the possible score range, a rule fixed by the instrument rather than derived from the observed distribution: low = 1.00–2.33, medium = 2.34–3.67, and high = 3.68–5.00. One deep-work item (“I can quickly shift my attention back to what I was doing after watching short-form videos”) is positively worded relative to the remaining eight items and was retained in its original scoring direction, as pre-specified in the pilot instrument; its corrected item-total correlation was 0.17, compared with 0.54–0.71 for the other items. A sensitivity analysis reverse-scoring this item is reported in the Results and did not alter any conclusion.

### Sample size

Two sample-size considerations were applied. First, for the descriptive objective, the single-population-proportion formula *n* = *Z*^2^*p* (1–*p*)/*d*^2^ was used with *Z* = 1.96 (95% confidence), an assumed proportion *p* = 0.50 (the value maximizing the required sample in the absence of a prior local estimate of SFV reward expectancy), and an absolute margin of error *d* = 0.05, giving *n* = 384. Inflating this by an anticipated 20% non-response using *n*/(1–0.20) gave a target of 480 participants. Second, because the primary analyses are association models rather than a prevalence estimate, an effect-size-based justification was also applied: for a multiple linear regression with 14 predictors, detecting a medium effect (*f* ^2^ = 0.15) with 80% power at α = 0.05 requires 135 participants, and detecting a small-to-moderate effect (*f* ^2^ = 0.05) requires approximately 395. The achieved analytic sample of 514 exceeds all of these thresholds. The observed effect sizes were large (*f* ^2^ = 0.72 for fragmentation and *f* ^2^ = 0.37 for deep-work impairment in the crude models). The study was not powered to detect effects smaller than *f* ^2^ ≈ 0.02 with the full covariate set, which would have required approximately 929 participants.

### Ethical considerations

Electronic informed consent was obtained from all participants before participation. No personally identifiable information was collected, and all data were stored securely and were accessible only to the research team. Findings were reported in aggregate form to maintain participant anonymity and confidentiality. Ethical approval was obtained from the Institutional Review Board (IRB) at King Saud University on 14 December 2025 (IRB approval number: E-25-10364), and all recruitment and data collection took place after that date.

### Statistical analysis

Data were analyzed using IBM SPSS Statistics, Version 21.0. Frequencies and percentages were used to describe categorical variables, and means and standard deviations to summarize the study scores. Confidence intervals for proportions were computed using the normal approximation. Internal consistency was assessed using Cronbach's alpha.

Bivariate associations between SFV reward expectancy and each outcome were examined using Pearson's correlation coefficient with Fisher z-transformed 95% confidence intervals. Because Likert-derived scores are strictly ordinal at the item level, Spearman's rank correlation was computed in parallel for every bivariate association; scale scores were treated as continuous in the regression models on the grounds that each is the mean of 8 to 11 items and approximates an interval scale, and the close agreement between the parametric and rank-based estimates is reported as support for this treatment.

Hierarchical multiple linear regression was used to estimate the association between SFV reward expectancy and each outcome with progressive adjustment. Model 1 was unadjusted. Model 2 added demographic and academic covariates: age group, sex, postgraduate status, self-reported BMI category (including a separate category for “don't know/prefer not to answer”), hours studied per session, and study location (home, library, university). Model 3 additionally adjusted for device-related study behaviors: frequency of phone or device use while studying, and self-reported multitasking while studying. Model 3 is presented as a conservative estimate, because these two behaviors are conceptually proximal to the fragmentation construct and may lie on the pathway between reward expectancy and fragmented studying rather than confounding it; adjustment for them may therefore represent over-adjustment.

For every model, unstandardized coefficients (B) with standard errors and 95% confidence intervals, standardized coefficients (β), *R*^2^, and adjusted *R*^2^ are reported. Collinearity was assessed using variance inflation factors (VIF) and tolerance. Linearity was assessed by inspection of residual-vs.-fitted plots and by adding a quadratic term for SFV reward expectancy; homoscedasticity was tested using the Breusch-Pagan test and, as a safeguard, heteroscedasticity-consistent (HC3) standard errors were computed for every model; normality of residuals was assessed by skewness, kurtosis, and normal probability plots; and independence of residuals was assessed using the Durbin-Watson statistic.

Two primary outcomes were pre-specified, giving two primary comparisons; a Bonferroni-adjusted threshold of α = 0.025 was therefore applied to the primary tests, both of which remained significant by a wide margin. Covariate coefficients within the adjusted models are reported as exploratory and were not corrected for multiplicity. Common-method variance was assessed using Harman's single-factor test across all 28 scale items. Statistical significance was set at *p* < 0.05 (two-tailed) unless otherwise stated.

The independent variable was SFV reward expectancy. The outcome variables were study-session fragmentation and deep-work impairment. Measures of academic workload, study-related stress, sleep duration, anxiety symptoms, and attention-deficit/hyperactivity disorder (ADHD) status were not collected in this study; hours studied per session and multitasking frequency were the only available proxies for academic load and are treated as such.

## Results

## Participant characteristics

A total of 514 participants were included in the analysis. The majority were aged 16–20 years (45.5%), followed by 21–25 years (37.9%). Males represented 53.3%, while females accounted for 46.7%. Most participants were pursuing a bachelor's degree (80.0%), with smaller proportions in master's (10.1%) and PhD (6.0%) programs. Regarding BMI, 50.2% had normal weight, while 19.3% were overweight and 8.6% were obese. In terms of study habits, 46.3% reported studying 1–2 h per study session, and most students studied at home (92.5%). Notably, 68.3% reported often or always using their phones while studying, and 57.8% agreed or strongly agreed that they multitask during study sessions (Tables 1, 2).

**Table 1:** Sociodemographic characteristics of participants (*N* = 514).

Characteristic	Frequency (*n*)	Percentage (%)
Gender	
Male	274	53.3
Female	240	46.7
Age (years)	
16–20	234	45.5
21–25	195	37.9
26–30	38	7.4
31–35	25	4.9
36–40	22	4.3
Academic level	
Diploma	20	3.9
Bachelor's degree	411	80.0
Master's degree	52	10.1
Doctorate (PhD)	31	6.0
BMI (self-reported)	
Underweight	42	8.2
Normal weight	258	50.2
Overweight	99	19.3
Obese	44	8.6
Don't know/prefer not to answer	71	13.8

Percentages are based on the analytic sample, *N* = 514.

BMI, body mass index.

All frequencies and percentages have been recalculated following removal of one duplicate submission.

**Table 2:** Study-related behaviors of participants (*N* = 514).

Characteristic	Frequency (*n*)	Percentage (%)
Hours studied per study session		
Less than 1 h	81	15.8
1–2 h	238	46.3
3–4 h	142	27.6
5 or more h	53	10.3
Study location (multiple response)		
Home	472	92.5
Library	90	17.6
Café	94	18.4
University	163	32.0
Phone/device use while studying		
Never	8	1.6
Rarely	37	7.2
Sometimes	118	23.0
Often	198	38.5
Always	153	29.8
Multitasking while studying		
Strongly disagree	32	6.2
Disagree	57	11.1
Neutral	128	24.9
Agree	185	36.0
Strongly agree	112	21.8

Percentages are based on the analytic sample, *N* = 514, except for study location.

Study location was a multiple-response item: respondents could select more than one option, so percentages sum to more than 100% and are expressed as percent of the 510 participants who selected at least one location.

The item was previously labeled “daily study hours” and is corrected here to “hours studied per study session,” which is the wording used in the questionnaire.

## Distribution of SFV reward expectancy

High SFV reward expectancy was reported by 58.4% of respondents in this sample (95% CI: 54.1–62.6%). A moderate level was reported by 33.1% and a low level by 8.6%. The mean SFV score was 3.76 ± 0.95. As noted in the Methods, these proportions describe the present voluntary-response sample and should not be read as prevalence estimates for the King Saud University student body or for Saudi university students more broadly (Table 3; Figure 1).

**Table 3:** Distribution of participants according to levels of the main study variables (*N* = 514).

Variable	Low, *n* (%)	Medium, *n* (%)	High, *n* (%)	Mean ±SD
SFV reward expectancy	44 (8.6)	170 (33.1)	300 (58.4)	3.76 ± 0.95
Study-session fragmentation	37 (7.2)	258 (50.2)	219 (42.6)	3.54 ± 0.84
Deep-work impairment	53 (10.3)	242 (47.1)	219 (42.6)	3.50 ± 0.78

Percentages are based on the analytic sample, *N* = 514.

SFV, short-form video. Levels were defined by equal thirds of the possible score range (low = 1.00–2.33; medium = 2.34–3.67; high = 3.68–5.00), a rule fixed by the instrument and not derived from the observed distribution. Higher deep-work impairment scores indicate greater self-reported difficulty sustaining concentration.

**Figure 1:**
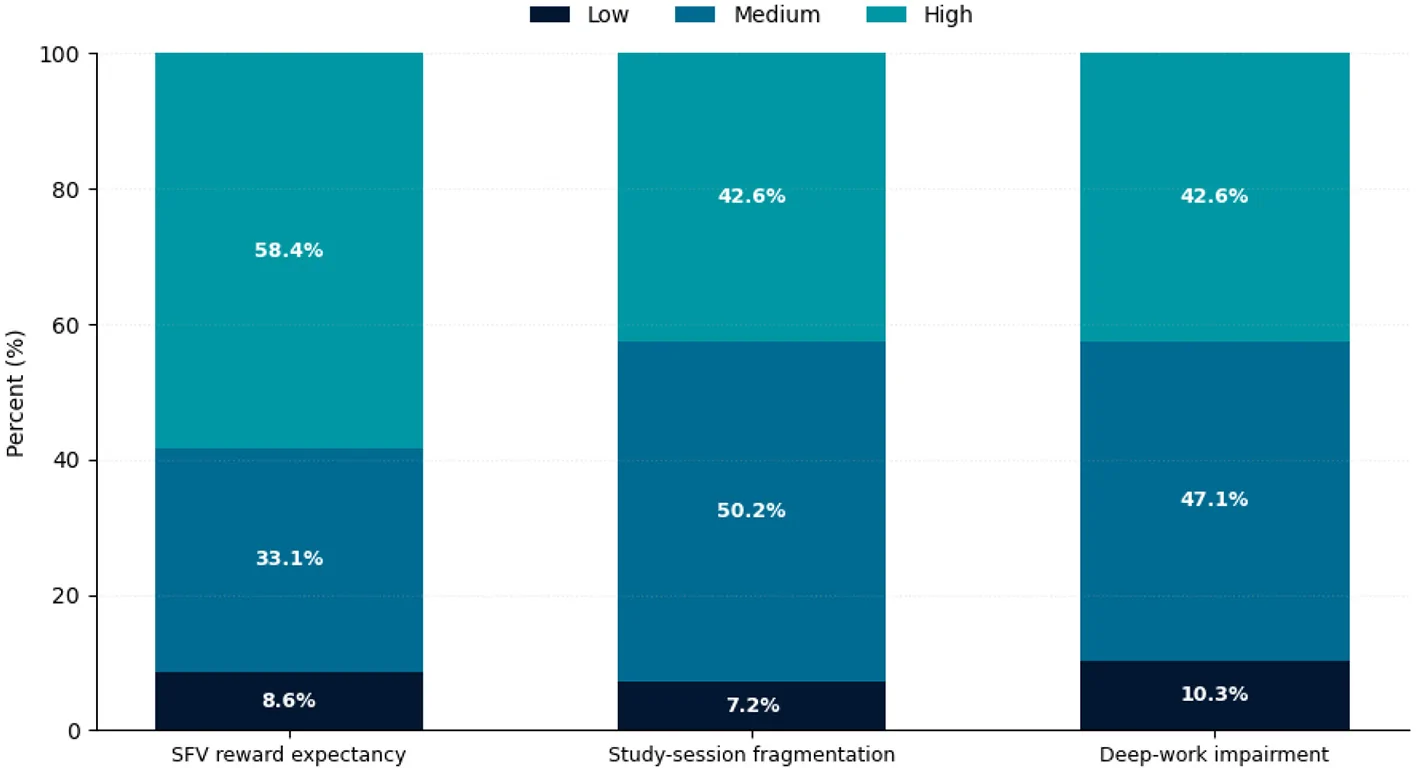
Distribution of participants according to levels of the main study variables. Bars show the percentage of participants classified as low, medium, or high for each main study variable (*N* = 514). SFV, short-form video. Levels reflect equal thirds of the possible score range.

## Association between SFV reward expectancy and study-session fragmentation

SFV reward expectancy was positively and significantly associated with study-session fragmentation (*r* = 0.646, 95% CI 0.593–0.694, *p* < 0.001; Spearman's ρ = 0.643, *p* < 0.001). In the unadjusted regression model, each one-point increase in SFV reward expectancy score corresponded to a 0.571-point higher fragmentation score (95% CI 0.513–0.630, SE = 0.030, β = 0.646, *p* < 0.001). The association was essentially unchanged after adjustment for demographic and academic characteristics (Model 2: B = 0.561, 95% CI 0.502–0.619, β = 0.634, *p* < 0.001) and remained substantial, although attenuated, after further adjustment for phone use and multitasking while studying (Model 3: *B* = 0.441, 95% CI 0.379–0.503, β = 0.499, *p* < 0.001). Adjusted R^2^ rose from 0.417 in Model 1 to 0.500 in Model 3. Among the covariates, more hours studied per session was associated with lower fragmentation (*B* = −0.084, *p* = 0.008), while more frequent phone use (*B* = 0.131, *p* < 0.001) and multitasking (*B* = 0.142, *p* < 0.001) were associated with higher fragmentation (Tables 4–7; Figure 2).

**Table 4:** Correlations between SFV reward expectancy and study-related outcomes (*N* = 514).

Outcome variable	Pearson's *r*	95% CI	Spearman's ρ	*p*-value
Study-session fragmentation	0.646	0.593–0.694	0.643	< 0.001
Deep-work impairment	0.518	0.452–0.578	0.524	< 0.001

The predictor in both rows is SFV reward expectancy.

Confidence intervals for r were obtained by Fisher z-transformation. Spearman's ρ is reported because Likert-derived scores are ordinal at item level; the close agreement between the two coefficients supports treating the scale means as continuous. p-values are identical to three decimal places for both coefficients.

SFV, short-form video.

**Table 5:** Unadjusted linear regression of study outcomes on SFV reward expectancy (*N* = 514).

Outcome variable	*B*	SE	95% CI	β	*R^2^*
Study-session fragmentation	0.571	0.030	0.513–0.630	0.646	0.418
Deep-work impairment	0.430	0.031	0.368–0.491	0.518	0.268

B, unstandardized regression coefficient; SE, standard error; β, standardized coefficient; *R*^2^, coefficient of determination.

All *p* < 0.001.

In a single-predictor model *R*^2^ is arithmetically equal to *r*^2^, so these models do not provide evidence independent of Table 4 and are retained only to express the association in the original scale units. Adjusted estimates are given in Table 6.

SFV, short-form video.

**Table 6:** Hierarchical multiple linear regression models for study-session fragmentation and deep-work impairment (*N* = 514).

Model	*B* (SFV)	SE	95% CI	β	Adj. *R*^2^	*p*
Study-session fragmentation						
Model 1-unadjusted	0.571	0.030	0.513–0.630	0.646	0.417	< 0.001
Model 2-+ demographic/academic	0.561	0.030	0.502–0.619	0.634	0.432	< 0.001
Model 3-+ device behavior	0.441	0.032	0.379–0.503	0.499	0.500	< 0.001
Deep-work impairment						
Model 1-unadjusted	0.430	0.031	0.368–0.491	0.518	0.267	< 0.001
Model 2-+ demographic/academic	0.423	0.032	0.361–0.486	0.510	0.270	< 0.001
Model 3-+ device behavior	0.357	0.035	0.288–0.426	0.431	0.295	< 0.001

Coefficients shown are for SFV reward expectancy.

Model 2 adjusts for age group, sex, postgraduate status, BMI category, hours studied per session, and study location (home, library, university).

Model 3 additionally adjusts for phone/device use while studying and multitasking while studying; because these behaviors are conceptually proximal to the fragmentation construct and may be intermediate rather than confounding variables, Model 3 is a conservative estimate and may over-adjust. Maximum VIF across models = 2.65 (all tolerances > 0.37).

Breusch-Pagan p = 0.11–0.27; HC3 robust standard errors did not alter any inference.

Durbin-Watson 1.98–2.04; residual skewness −0.23 to −0.29, kurtosis −0.01 to 0.31.

B, unstandardized coefficient; β, standardized coefficient; SFV, short-form video.

**Table 7:** Covariates independently associated with each outcome in the fully adjusted models (*N* = 514).

Outcome	Covariate	*B*	SE	*p*-value
Study-session fragmentation	Hours studied per session	−0.084	0.031	0.008
	Phone/device use while studying	0.131	0.034	< 0.001
	Multitasking while studying	0.142	0.029	< 0.001
Deep-work impairment	Multitasking while studying	0.108	0.032	0.001

Only covariates reaching *p* < 0.05 in Model 3 are shown; full model output is available in Supplementary File 2. Age, sex, postgraduate status, BMI category, and study location were not independently associated with either outcome. These covariate estimates are exploratory and were not corrected for multiplicity.

**Figure 2:**
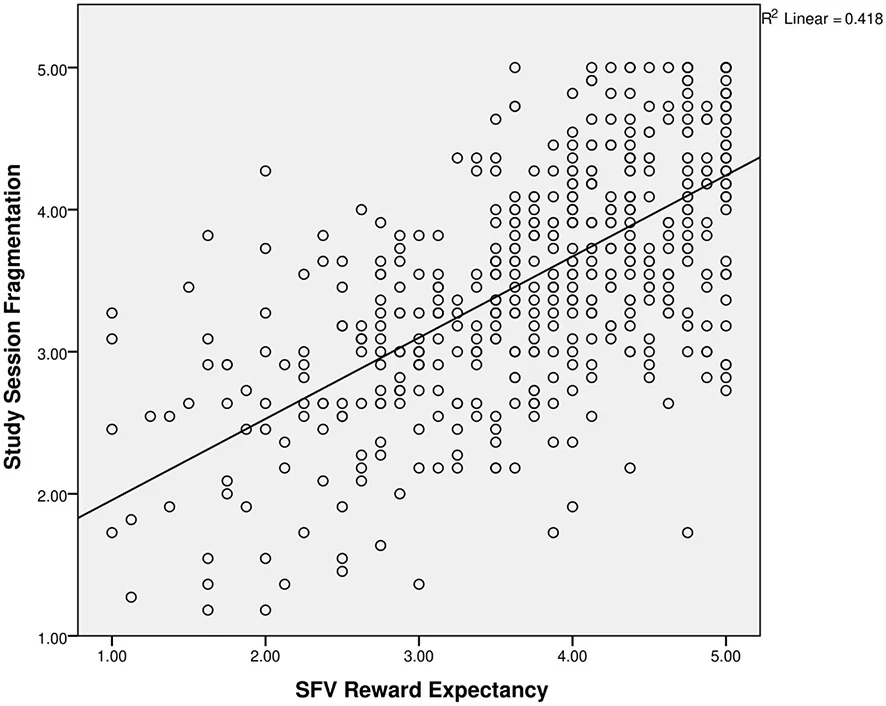
Relationship between SFV reward expectancy and study-session fragmentation. Pearson's *r* = 0.646; *R*^2^ = 0.418; *N* = 514.

## Association between SFV reward expectancy and deep-work impairment

SFV reward expectancy was also positively and significantly associated with deep-work impairment (*r* = 0.518, 95% CI 0.452–0.578, *p* < 0.001; Spearman's ρ = 0.524, *p* < 0.001), indicating that students reporting higher reward expectancy also reported greater difficulty sustaining concentration on demanding academic tasks. In the unadjusted model, each one-point increase in SFV reward expectancy corresponded to a 0.430-point higher deep-work impairment score (95% CI 0.368–0.491, SE = 0.031, β = 0.518, *p* < 0.001). The association persisted after adjustment for demographic and academic characteristics (Model 2: *B* = 0.423, 95% CI 0.361–0.486, β = 0.510, *p* < 0.001) and after additional adjustment for device-related study behaviors (Model 3: *B* = 0.357, 95% CI 0.288–0.426, β = 0.431, *p* < 0.001). No demographic or academic covariate was independently associated with deep-work impairment; among device behaviors, only multitasking was (*B* = 0.108, *p* = 0.001) (Tables 4–7; Figure 3).

**Figure 3:**
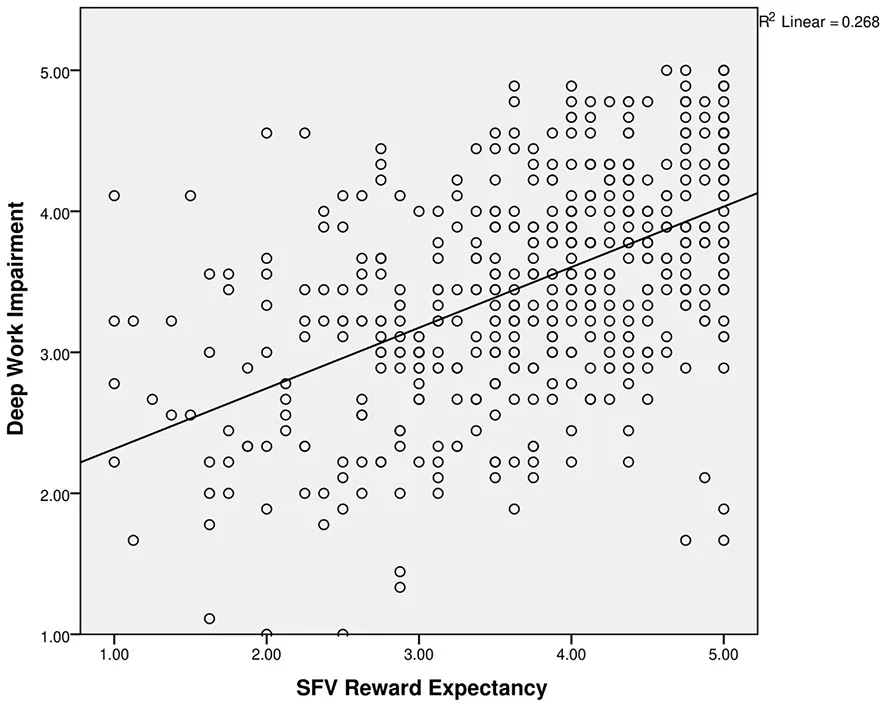
Relationship between SFV reward expectancy and deep-work impairment. Pearson's *r* = 0.518; *R*^2^ = 0.268; *N* = 514. Higher scores on the *y*-axis indicate greater self-reported deep-work impairment.

## Model diagnostics and sensitivity analyses

Regression assumptions were satisfactory in all models. Variance inflation factors did not exceed 2.65 (all tolerances > 0.37), indicating no problematic collinearity. Breusch-Pagan tests were non-significant for all adjusted models (*p* = 0.11–0.27); HC3 heteroscedasticity-consistent standard errors differed from ordinary least squares standard errors by no more than 0.005 and did not alter any inference. Residuals were approximately normally distributed (skewness −0.23 to −0.29; kurtosis −0.01 to 0.31) and independent (Durbin-Watson 1.98–2.04). Adding a quadratic term for SFV reward expectancy produced a statistically detectable but negligible improvement in fit (Δ*R*^2^ = 0.006 for fragmentation and 0.009 for deep-work impairment), so linear models were retained.

Two sensitivity analyses were performed. Reverse-scoring the single positively-worded deep-work item yielded a marginally stronger association (*r* = 0.534; *B* = 0.419, *R*^2^ = 0.285) and did not change any conclusion. Harman's single-factor test across all 28 items showed that the first unrotated factor accounted for 40.3% of the total variance, below the conventional 50% threshold, indicating that common-method variance is unlikely to account for the observed associations, although it cannot be excluded.

## Discussion

This study found that high SFV reward expectancy was reported by more than half of the participants (58.4%), indicating that reward-driven engagement with platforms such as TikTok, Instagram Reels, and YouTube Shorts was common in this sample. Higher SFV reward expectancy was significantly associated with greater study-session fragmentation and with greater self-reported deep-work impairment, and both associations persisted after adjustment for demographic, academic, and device-use characteristics. Students who more strongly anticipated quick rewards from SFV content therefore also reported more interrupted study sessions and greater difficulty sustaining academic focus.

The high proportion observed in this study is broadly consistent with previous international and regional studies showing widespread engagement with short-form video platforms among young people and university students (Jain et al., 2025; Ye et al., 2022; Guo and Chai, 2024). Similar concerns have also been reported in Saudi Arabia, where SFV use has been associated with reduced attention, memory difficulties, and cognitive fatigue among social media users (Alruwaili, 2025). However, the present study extends previous findings by specifically examining reward expectancy and its relationship with fragmented studying behaviors and deep-work impairment within a Saudi university context. This pattern may be partly explained by the design of SFV platforms, which rely on short, personalized, and rapidly changing content that encourages repeated viewing and reward-seeking behavior (Zhao and Wagner, 2022; Zhao, 2021). Among university students, frequent smartphone use, high social media engagement, and the growing integration of digital platforms into daily routines may further contribute to elevated SFV reward expectancy.

A major finding of this study was the association between SFV reward expectancy and study-session fragmentation (*r* = 0.646; adjusted *B* = 0.441, 95% CI 0.379–0.503, *p* < 0.001). This indicates that students with stronger reward expectancy toward SFV content are more likely to report interrupted study sessions, phone checking, and multitasking. These findings are consistent with previous studies showing that excessive SFV use is associated with impaired attention control, impulsivity, weaker inhibitory control, and learning avoidance (Yan et al., 2024; Chen et al., 2022; Ye et al., 2023). One behavioral interpretation, which the present data can support only as a hypothesis, is that repeated exposure to rapid digital rewards renders sustained academic tasks comparatively less stimulating and increases the tendency to switch attention during studying.

The present study also found a significant association between SFV reward expectancy and deep-work impairment (*r* = 0.518; adjusted *B* = 0.357, 95% CI 0.288–0.426, *p* < 0.001), such that higher SFV reward expectancy was associated with greater self-reported difficulty sustaining concentration. This interpretation is consistent with neurocognitive evidence signifying that excessive SFV exposure may be accompanied by differences in executive functioning and sustained attention (Yan et al., 2024; Chen et al., 2022; Gao et al., 2025), and with neuroimaging research showing activation of reward-processing regions, including the ventral tegmental area and default mode network, during exposure to personalized TikTok content (Su et al., 2021; Li et al., 2025). It must be noted that no neurocognitive, imaging, or behavioral performance measures were obtained in the present study. These mechanisms are therefore offered as hypotheses generated by prior experimental work to be tested in future research, not as processes showed by the present data, which consist entirely of concurrent self-report.

The observed relationships should be regarded as bidirectional in principle. While high SFV reward expectancy may contribute to fragmented studying and impaired sustained concentration, students with pre-existing attentional difficulties may equally be drawn to highly stimulating SFV content, and a third factor may contribute to both. The variance explained by SFV reward expectancy (adjusted *R*^2^ = 0.500 for fragmentation and 0.295 for deep-work impairment in the full models) should be read as the proportion of variance accounted for within these specific models and covariate sets, not as an independent or causal contribution; the crude values of 41.8% and 26.8% in particular derive from single-predictor models and necessarily overstate the unique contribution of reward expectancy.

## Strengths

The study addressed a locally relevant topic within the Saudi university setting, where short-form video use may have important implications for students' academic behaviors and concentration. The use of internally reliable scales supports the consistency of the measured constructs. In addition, by examining both study-session fragmentation and deep-work impairment, the study provides a broader understanding of how short-form video reward expectancy may be associated with students' academic focus and learning-related behaviors. The sample size was larger than required for the analyses performed, and the association was robust to progressive covariate adjustment, to rank-based estimation, to heteroscedasticity-consistent standard errors, and to alternative scoring of the deep-work scale.

## Implications

These findings are associative and should be viewed as directions for future intervention research rather than validated recommendations. Educational programmes could focus on digital wellness, attention management, and reducing multitasking during study, as study-related behaviors may be more relevant intervention targets than SFV use alone (Lin et al., 2020). In university health settings, students with persistent concentration difficulties may permit consideration of their digital-media use alongside established causes, although the present study did not assess clinical conditions or support screening recommendations (Li, 2024). At the institutional level, universities may consider incorporating digital literacy and healthy technology-use guidance into student support services and creating study environments that facilitate uninterrupted work. Such interventions and policies should be evaluated prospectively before wider implementation (Guo and Chai, 2024).

## Limitations

Several limitations should be considered. First, the cross-sectional design prevents establishing temporal or causal relationships between short-form video reward expectancy, study-session fragmentation, and deep-work impairment, or assessing long-term consequences. Second, self-reported data may be affected by recall and social desirability bias and may not reflect actual video use or study interruptions. Because all constructs were measured using the same instrument at one sitting, common-method bias is also possible; however, Harman's single-factor test showed 40.3% variance in the first unrotated factor, meaning it was unlikely to be the main explanation. Third, voluntary-response sampling may have introduced selection bias, and the number of students invited was not recorded, preventing calculation of a response rate. Thus, findings should be interpreted as sample characteristics rather than population estimates. Fourth, although the adapted instrument showed internal consistency, its structural validity remains unconfirmed; no factor analysis was performed, and one deep-work item had a weak corrected item-total correlation (*r* = 0.17), without affecting the results when removed or reverse-scored. Fifth, important potential confounders, including reward sensitivity, ADHD symptoms, anxiety/depression, sleep, smartphone screen time, academic workload, and study-related stress, were not assessed, so residual confounding cannot be excluded. Finally, the single-institution design limits generalizability. Future multicentre longitudinal studies using validated individual-difference measures and objective measures of attention and device use are needed to clarify causality and evaluate interventions.

## Conclusion

This study provides local evidence on the relationship between short-form video (SFV) reward expectancy, study-session fragmentation, and self-reported deep-work impairment among King Saud University students. High SFV reward expectancy was commonly reported in this sample and was significantly associated with greater study-session fragmentation and greater deep-work impairment; both associations were weakened but remained significant after adjustment for demographic, academic, and device-use characteristics. Students reporting higher SFV reward expectancy also reported more interrupted study sessions, more frequent multitasking, and greater difficulty maintaining concentration on cognitively demanding academic tasks. Because the design is cross-sectional and all measures were self-reported, these findings identify an association of academic and cognitive relevance but cannot establish that SFV engagement causes fragmented studying or impaired concentration. Longitudinal and multi-center studies incorporating objective measures of attention and device use, and validated measures of reward sensitivity and ADHD symptoms, are needed before intervention strategies can be recommended.

## Data Availability

The raw data supporting the conclusions of this article will be made available by the authors, without undue reservation.
