# Supplementary material for: Short-form video reward expectancy, study-session fragmentation, and self-reported deep-work impairment among King Saud University students: a cross-sectional study
Source: Front Psychol. 2026 Oct 30;17:1969859. doi: 10.3389/fpsyg.2026.1969859 (PMC13759777; doi:10.3389/fpsyg.2026.1969859)
Supplement: Supplementary file 1 [file Supplementary_file_1.docx]

**Association between Short-form Video (TikTok, Instagram Reels, YouTube Shorts) Reward-Expectancy and Study Session Fragmentation and Deep Work Impairment among University Students**

You are invited to participate in this survey, and your contribution is highly valued as it will help us achieve the intended purpose of this study.

Study Title: Association between Short-form Video (TikTok, Instagram Reels, YouTube Shorts) Reward-Expectancy and Study Session Fragmentation and Deep Work impairment among King Saud University Students.

The purpose of this online survey is to examine the relationship between the use of short-form videos and the reward expectancy associated with them, as well as their impact on study session fragmentation and deep focus ability among King Saud University students. The importance of this study lies in understanding the effect of digital behavior on concentration quality and academic performance, thereby contributing to raising awareness of healthy study habits.

This survey will take approximately 5–7 minutes to complete. Your participation is completely voluntary, and you may withdraw at any time. Responses are anonymous and confidential, and the researcher will not be able to identify participants. You will not be asked for your name or any identifying information. The collected data will be used solely for research purposes.

For any questions, please contact Dr. Mussad Bedah Alsahly at ([Musad310@gmail.com](mailto:Musad310@gmail.com)).

Do you agree to participate voluntarily in this anonymous survey?

☐ Yes, I agree

☐ No, I do not agree (If you select “No,” the survey will end.)

(1) Eligibility

1. Are you currently a student at King Saud University?

☐ Yes

☐ No (If you select “No,” you are not eligible to participate in this survey.)

(2) Demographic Information

1. Age group:

☐ 16–20 1

☐ 21–25 2

☐ 26–30 3

☐ 31–35 4

☐ 36–40 5

2. Gender:

☐ Male 1

☐ Female 2

3. College / Academic Program: (Select your current college or program)

☐ First Common Year (Preparatory Year Program) 1

☐ College of Medicine 2

☐ College of Dentistry 3

☐ College of Nursing 4

☐ College of Applied Medical Sciences 5

☐ College of Pharmacy 6

☐ College of Science 7

☐ College of Engineering 8

☐ College of Computer and Information Sciences 9

☐ College of Business Administration 10

☐ College of Education 11

☐ College of Arts 12

☐ College of Law and Political Science 13

☐ College of Languages and Translation 14

☐ College of Agriculture and Food Sciences 15

☐ College of Architecture and Planning 16

☐ College of Tourism and Antiquities 17

☐ College of Sport Sciences and Physical Activity 18

☐ College of Applied Studies and Community service 19

☐ Prince Sultan bin Abdulaziz College for Emergency Medical Services 20

☐ College of Humanities and Social Science 21

4. What academic level are you currently pursuing?

☐ Diploma 1

☐ Bachelor’s degree 2

☐ Master’s degree 3

☐ Doctorate (PhD) 4

5. BMI Category (Self-reported):

You can check your BMI using any online BMI calculator if unsure.)

☐ Underweight (BMI < 18.5) 1

☐ Normal weight (BMI 18.5–24.9) 2

☐ Overweight (BMI 25–29.9) 3

☐ Obese (BMI ≥ 30) 4

☐ I don’t know / prefer not to answer 5

(3) Lifestyle & Study Habits

1. How many hours do you usually study per session?

☐ Less than 1 hour 1

☐ 1–2 hours 2

☐ 3–4 hours 3

☐ 5 or more hours 4

2. Where do you usually study?

☐ Home 1

☐ Library 2

☐ Café 3

☐ University 4

☐ Other: ___________

3. How often do you use your phone or digital devices while studying?

☐ Never 1

☐ Rarely 2

☐ Sometimes 3

☐ Often 4

☐ Always 5

4. I usually multitask when studying (e.g., switching between apps or checking social media).

☐ Strongly Disagree 1

☐ Disagree 2

☐ Neutral 3

☐ Agree 4

☐ Strongly Agree 5

(4) Short-form Video Exposure & Reward-Expectancy

1. I check short-form video platforms (TikTok, Instagram Reels) several times a day.

☐ Strongly Disagree 1

☐ Disagree 2

☐ Neutral 3

☐ Agree 4

☐ Strongly Agree 5

2. My daily use of short-form videos has increased over the past year.

☐ Strongly Disagree 1

☐ Disagree 2

☐ Neutral 3

☐ Agree 4

☐ Strongly Agree 5

3. I feel a strong urge to watch short videos when I’m bored or idle.

☐ Strongly Disagree 1

☐ Disagree 2

☐ Neutral 3

☐ Agree 4

☐ Strongly Agree 5

4. Scrolling through short-form videos has become a routine habit.

☐ Strongly Disagree 1

☐ Disagree 2

☐ Neutral 3

☐ Agree 4

☐ Strongly Agree 5

5. I often watch short videos to feel happier.

☐ Strongly Disagree 1

☐ Disagree 2

☐ Neutral 3

☐ Agree 4

☐ Strongly Agree 5

6. I often need to watch short videos to forget my troubles.

☐ Strongly Disagree 1

☐ Disagree 2

☐ Neutral 3

☐ Agree 4

☐ Strongly Agree 5

7. I often need to watch short videos to ease my anxiety.

☐ Strongly Disagree 1

☐ Disagree 2

☐ Neutral 3

☐ Agree 4

☐ Strongly Agree 5

8. I often need to watch short videos to experience quick satisfaction.

☐ Strongly Disagree 1

☐ Disagree 2

☐ Neutral 3

☐ Agree 4

☐ Strongly Agree 5

(5) Study Session Fragmentation

1. I often study in short bursts rather than for long continuous periods.

☐ Strongly Disagree 1

☐ Disagree 2

☐ Neutral 3

☐ Agree 4

☐ Strongly Agree 4

2. I find it difficult to maintain focus for a long time when studying.

☐ Strongly Disagree 1

☐ Disagree 2

☐ Neutral 3

☐ Agree 4

☐ Strongly Agree 5

3. I often forget what I was doing after checking my phone or watching short videos.

☐ Strongly Disagree 1

☐ Disagree 2

☐ Neutral 3

☐ Agree 4

☐ Strongly Agree 5

4. I often have to reread material because my attention drifts away.

☐ Strongly Disagree 1

☐ Disagree 2

☐ Neutral 3

☐ Agree 4

☐ Strongly Agree 5

5. I often use short breaks during study to check short videos.

☐ Strongly Disagree 1

☐ Disagree 2

☐ Neutral 3

☐ Agree 4

☐ Strongly Agree 5

6. I often interrupt my study to watch short videos.

☐ Strongly Disagree 1

☐ Disagree 2

☐ Neutral 3

☐ Agree 4

☐ Strongly Agree 5

7. I can’t stop watching short videos, even when I’m trying to study.

☐ Strongly Disagree 1

☐ Disagree 2

☐ Neutral 3

☐ Agree 4

☐ Strongly Agree 5

8. Watching short videos reduces the quality of my study sessions.

☐ Strongly Disagree 1

☐ Disagree 2

☐ Neutral 3

☐ Agree 4

☐ Strongly Agree 5

9. Watching short videos makes it harder for me to return to studying afterward.

☐ Strongly Disagree 1

☐ Disagree 2

☐ Neutral 3

☐ Agree 4

☐ Strongly Agree 5

10. The more time I spend watching short videos, the greater it reduces my study efficiency.

☐ Strongly Disagree 1

☐ Disagree 2

☐ Neutral 3

☐ Agree 4

☐ Strongly Agree 5

11. I find myself switching between study tasks frequently.

☐ Strongly Disagree 1

☐ Disagree 2

☐ Neutral 3

☐ Agree 4

☐ Strongly Agree 5

(6) Deep Work impairment

1. After watching short-form videos, I find it harder to stay focused on tasks.

☐ Strongly Disagree 1

☐ Disagree 2

☐ Neutral 3

☐ Agree 4

☐ Strongly Agree 5

2. I get distracted more easily during study or work sessions.

☐ Strongly Disagree 1

☐ Disagree 2

☐ Neutral 3

☐ Agree 4

☐ Strongly Agree 5

3. I tend to switch tasks more frequently after using short video apps.

☐ Strongly Disagree 1

☐ Disagree 2

☐ Neutral 3

☐ Agree 4

☐ Strongly Agree 5

4. I struggle to concentrate on reading or listening for long periods.

☐ Strongly Disagree 1

☐ Disagree 2

☐ Neutral 3

☐ Agree 4

☐ Strongly Agree 5

5. I feel mentally tired after long sessions of scrolling through short videos.

☐ Strongly Disagree 1

☐ Disagree 2

☐ Neutral 3

☐ Agree 4

☐ Strongly Agree 5

6. My brain feels sluggish after using short-form video platforms.

☐ Strongly Disagree 1

☐ Disagree 2

☐ Neutral 3

☐ Agree 4

☐ Strongly Agree 5

7. I often feel mentally drained, even if I haven't done anything demanding.

☐ Strongly Disagree 1

☐ Disagree 2

☐ Neutral 3

☐ Agree 4

☐ Strongly Agree 5

8. After scrolling for a long time, I lose interest in doing productive tasks.

☐ Strongly Disagree 1

☐ Disagree 2

☐ Neutral 3

☐ Agree 4

☐ Strongly Agree 5

9. I can quickly shift my attention back to what I was doing after distractions.(R)

☐ Strongly Disagree 5

☐ Disagree 4

☐ Neutral 3

☐ Agree 2

☐ Strongly Agree 1

(7) Thank You Note

Thank you for your valuable time and honest responses.

Your participation will help us better understand how short-form video use affects attention, focus, and study habits among university students.

All data are collected anonymously and will be analyzed solely for research purposes.
