# Supplementary material for: Short-form video reward expectancy, study-session fragmentation, and self-reported deep-work impairment among King Saud University students: a cross-sectional study
Source: Front Psychol. 2026 Oct 30;17:1969859. doi: 10.3389/fpsyg.2026.1969859 (PMC13759777; doi:10.3389/fpsyg.2026.1969859)
Supplement: Supplementary file 2 [file Supplementary_file_2.docx]

**Supplementary File 2. Full multivariable regression output**

All models are ordinary least squares linear regressions. B = unstandardized coefficient; SE = standard error; β = standardized coefficient; VIF = variance inflation factor; Tol. = tolerance. Reference categories: male sex, undergraduate level, normal-weight BMI. Study location variables are binary indicators from a multiple-response item.

**S2.1 Study-session fragmentation — Model 2 (demographic and academic covariates)**

| **Predictor** | **B** | **SE** | **95% CI** | **β** | **p** | **VIF** | **Tol.** |
| --- | --- | --- | --- | --- | --- | --- | --- |
| SFV reward expectancy | 0.561 | 0.030 | 0.502 to 0.619 | 0.634 | <0.001 | 1.03 | 0.97 |
| Age group (ordinal) | 0.023 | 0.043 | -0.062 to 0.107 | 0.028 | 0.602 | 2.62 | 0.38 |
| Female sex | 0.031 | 0.060 | -0.087 to 0.149 | 0.019 | 0.608 | 1.17 | 0.85 |
| Postgraduate level | -0.134 | 0.123 | -0.375 to 0.108 | -0.059 | 0.277 | 2.65 | 0.38 |
| BMI: underweight | 0.094 | 0.105 | -0.113 to 0.300 | 0.031 | 0.373 | 1.08 | 0.93 |
| BMI: overweight | -0.010 | 0.077 | -0.162 to 0.142 | -0.005 | 0.898 | 1.21 | 0.83 |
| BMI: obese | 0.085 | 0.105 | -0.122 to 0.292 | 0.029 | 0.418 | 1.13 | 0.89 |
| BMI: don't know | -0.212 | 0.085 | -0.378 to -0.045 | -0.087 | 0.013 | 1.11 | 0.90 |
| Hours studied per session | -0.110 | 0.033 | -0.175 to -0.045 | -0.113 | <0.001 | 1.05 | 0.95 |
| Studies at home | 0.056 | 0.108 | -0.156 to 0.268 | 0.018 | 0.603 | 1.14 | 0.88 |
| Studies at library | 0.070 | 0.077 | -0.082 to 0.222 | 0.032 | 0.363 | 1.12 | 0.89 |
| Studies at university | 0.070 | 0.064 | -0.055 to 0.195 | 0.039 | 0.271 | 1.14 | 0.88 |

*Model fit: R² = 0.446; adjusted R² = 0.432; F(12, 501) = 33.58, p < 0.001*

**S2.2 Study-session fragmentation — Model 3 (adding device-related study behaviours)**

| **Predictor** | **B** | **SE** | **95% CI** | **β** | **p** | **VIF** | **Tol.** |
| --- | --- | --- | --- | --- | --- | --- | --- |
| SFV reward expectancy | 0.441 | 0.032 | 0.379 to 0.503 | 0.499 | <0.001 | 1.31 | 0.76 |
| Age group (ordinal) | 0.018 | 0.040 | -0.062 to 0.097 | 0.022 | 0.660 | 2.62 | 0.38 |
| Female sex | -0.005 | 0.057 | -0.117 to 0.107 | -0.003 | 0.930 | 1.18 | 0.85 |
| Postgraduate level | -0.139 | 0.115 | -0.365 to 0.088 | -0.061 | 0.230 | 2.65 | 0.38 |
| BMI: underweight | 0.046 | 0.099 | -0.148 to 0.241 | 0.015 | 0.639 | 1.08 | 0.93 |
| BMI: overweight | -0.017 | 0.073 | -0.160 to 0.126 | -0.008 | 0.819 | 1.21 | 0.82 |
| BMI: obese | 0.004 | 0.099 | -0.191 to 0.200 | 0.001 | 0.965 | 1.14 | 0.88 |
| BMI: don't know | -0.188 | 0.080 | -0.344 to -0.031 | -0.078 | 0.019 | 1.11 | 0.90 |
| Hours studied per session | -0.084 | 0.031 | -0.145 to -0.022 | -0.086 | 0.008 | 1.06 | 0.94 |
| Studies at home | -0.063 | 0.102 | -0.265 to 0.138 | -0.021 | 0.537 | 1.16 | 0.86 |
| Studies at library | 0.102 | 0.073 | -0.041 to 0.244 | 0.046 | 0.164 | 1.12 | 0.89 |
| Studies at university | 0.056 | 0.060 | -0.062 to 0.173 | 0.031 | 0.352 | 1.14 | 0.88 |
| Phone use while studying | 0.131 | 0.034 | 0.065 to 0.197 | 0.152 | <0.001 | 1.57 | 0.64 |
| Multitasking while studying | 0.142 | 0.029 | 0.086 to 0.198 | 0.192 | <0.001 | 1.54 | 0.65 |

*Model fit: R² = 0.513; adjusted R² = 0.500; F(14, 499) = 37.60, p < 0.001*

**S2.3 Deep-work impairment — Model 2 (demographic and academic covariates)**

| **Predictor** | **B** | **SE** | **95% CI** | **β** | **p** | **VIF** | **Tol.** |
| --- | --- | --- | --- | --- | --- | --- | --- |
| SFV reward expectancy | 0.423 | 0.032 | 0.361 to 0.486 | 0.510 | <0.001 | 1.03 | 0.97 |
| Age group (ordinal) | 0.031 | 0.046 | -0.059 to 0.121 | 0.041 | 0.499 | 2.62 | 0.38 |
| Female sex | 0.089 | 0.064 | -0.037 to 0.215 | 0.057 | 0.165 | 1.17 | 0.85 |
| Postgraduate level | -0.127 | 0.131 | -0.383 to 0.130 | -0.059 | 0.333 | 2.65 | 0.38 |
| BMI: underweight | 0.148 | 0.112 | -0.072 to 0.368 | 0.052 | 0.187 | 1.08 | 0.93 |
| BMI: overweight | 0.121 | 0.082 | -0.042 to 0.283 | 0.061 | 0.145 | 1.21 | 0.83 |
| BMI: obese | 0.020 | 0.112 | -0.200 to 0.240 | 0.007 | 0.859 | 1.13 | 0.89 |
| BMI: don't know | -0.161 | 0.090 | -0.338 to 0.016 | -0.071 | 0.075 | 1.11 | 0.90 |
| Hours studied per session | -0.038 | 0.035 | -0.107 to 0.031 | -0.042 | 0.282 | 1.05 | 0.95 |
| Studies at home | 0.013 | 0.115 | -0.213 to 0.239 | 0.005 | 0.909 | 1.14 | 0.88 |
| Studies at library | 0.084 | 0.082 | -0.078 to 0.246 | 0.041 | 0.308 | 1.12 | 0.89 |
| Studies at university | 0.036 | 0.068 | -0.097 to 0.169 | 0.021 | 0.598 | 1.14 | 0.88 |

*Model fit: R² = 0.288; adjusted R² = 0.270; F(12, 501) = 16.85, p < 0.001*

**S2.4 Deep-work impairment — Model 3 (adding device-related study behaviours)**

| **Predictor** | **B** | **SE** | **95% CI** | **β** | **p** | **VIF** | **Tol.** |
| --- | --- | --- | --- | --- | --- | --- | --- |
| SFV reward expectancy | 0.357 | 0.035 | 0.288 to 0.426 | 0.431 | <0.001 | 1.31 | 0.76 |
| Age group (ordinal) | 0.028 | 0.045 | -0.061 to 0.117 | 0.037 | 0.536 | 2.62 | 0.38 |
| Female sex | 0.066 | 0.063 | -0.058 to 0.191 | 0.042 | 0.296 | 1.18 | 0.85 |
| Postgraduate level | -0.132 | 0.129 | -0.384 to 0.121 | -0.062 | 0.306 | 2.65 | 0.38 |
| BMI: underweight | 0.124 | 0.110 | -0.093 to 0.341 | 0.043 | 0.261 | 1.08 | 0.93 |
| BMI: overweight | 0.114 | 0.081 | -0.045 to 0.273 | 0.057 | 0.161 | 1.21 | 0.82 |
| BMI: obese | -0.028 | 0.111 | -0.246 to 0.189 | -0.010 | 0.797 | 1.14 | 0.88 |
| BMI: don't know | -0.147 | 0.089 | -0.322 to 0.027 | -0.065 | 0.097 | 1.11 | 0.90 |
| Hours studied per session | -0.022 | 0.035 | -0.091 to 0.046 | -0.025 | 0.521 | 1.06 | 0.94 |
| Studies at home | -0.058 | 0.114 | -0.283 to 0.166 | -0.020 | 0.609 | 1.16 | 0.86 |
| Studies at library | 0.099 | 0.081 | -0.060 to 0.258 | 0.048 | 0.222 | 1.12 | 0.89 |
| Studies at university | 0.027 | 0.067 | -0.104 to 0.158 | 0.016 | 0.685 | 1.14 | 0.88 |
| Phone use while studying | 0.041 | 0.038 | -0.033 to 0.115 | 0.051 | 0.276 | 1.57 | 0.64 |
| Multitasking while studying | 0.108 | 0.032 | 0.045 to 0.171 | 0.156 | <0.001 | 1.54 | 0.65 |

*Model fit: R² = 0.314; adjusted R² = 0.295; F(14, 499) = 16.34, p < 0.001*

Diagnostics (all models): maximum VIF = 2.65, all tolerances > 0.37; Breusch–Pagan p = 0.11–0.27; HC3 heteroscedasticity-consistent standard errors differed from OLS standard errors by ≤ 0.005 and altered no inference; Durbin–Watson 1.98–2.04; residual skewness −0.23 to −0.29 and kurtosis −0.01 to 0.31. Adding a quadratic term for SFV reward expectancy increased R² by 0.006 (fragmentation) and 0.009 (deep-work impairment).

Common-method variance: Harman's single-factor test across all 28 scale items gave a first unrotated factor accounting for 40.3% of total variance (five eigenvalues > 1), below the conventional 50% threshold.
